# The Complex Etiology of Childhood Obesity in Arabs Is Highlighted by a Combination of Biological and Socio-Economic Factors

**DOI:** 10.3389/fpubh.2019.00072

**Published:** 2019-04-02

**Authors:** Naser Elkum, Monira Alarouj, Abdullah Bennakhi, Azza Shaltout

**Affiliations:** ^1^Research Department, Sidra Medicine, Doha, Qatar; ^2^Department of Clinical Services, Dasman Diabetes Institute, Kuwait City, Kuwait

**Keywords:** obesity, children, Arabs, prevalence, risk factors

## Abstract

**Objectives:** To identify predictors of childhood and adolescent obesity in Kuwaitis with Arab ethnicity.

**Methods:** A cross-sectional sample of 6–18 year-old schoolchildren was randomly selected from 244 public schools across all six governorates in the State of Kuwait. Anthropometric data were measured from 6,574 Arab Kuwaiti schoolchildren, and a structured questionnaire was used to collect information on possible risk factors associated with obesity. Overweight and obesity were defined in accordance with the Center for Disease Control and Prevention criteria.

**Results:** The prevalence of overweight and obesity in children (aged 6–18 years) were 17.7% and 33.7%, respectively. The likelihood of childhood obesity increased with birth weights >4.0 Kg [odds ratio (OR) = 2.3; *p* < 0.0001], maternal employment (OR = 1.26, *p* = 0.0006), maternal age at pregnancy >30 years (OR = 1.24; *p* = 0.0016) and family size of <6 members (OR = 1.16, *p* = 0.0106).

**Conclusions:** Public health professionals should be aware that advanced maternal age, maternal employment, smaller family size, and high birthweight may predict the risk of obesity in Kuwaiti Arab children and adolescents.

## Introduction

Childhood obesity is one of the most serious public health challenges worldwide. It has been shown that progression of obesity from childhood to adulthood is associated with the development of type 2 diabetes (T2D) (1, 2) and cardiovascular diseases (CVDs) (3). Our previous report (4) has demonstrated a markedly high prevalence of childhood obesity in Kuwait that exceeded those reported in neighboring countries and North America. This public health crisis requires a serious investigation to identify the key factors contributing to the high prevalence of childhood obesity in Kuwait.

A number of childhood obesity-related etiological factors have been identified, such as genetic, environmental and socio-economic risk factors (5–7). In addition, it is becoming increasingly evident that birthweight, short sleep duration, physical inactivity, parental obesity, and parental income are important predictive factors of childhood overweight and obesity in various countries. However, the majority of these factors were identified and their effects were studied only in Western populations. Therefore, in order to design meaningful prevention strategies, it is important to identify the contributing factors specific to each population and geographical location and to understand the reasons for the observed differences.

Few studies have investigated the factors associated with the prevalence of overweight and obesity in Kuwait, but differences in methodologies and target populations make comparison between these studies difficult. Improving our understanding of the factors associated with childhood overweight and obesity is crucial not only to improve child health but also to curb the increasing rates of adult obesity and its complications, such as T2D and CVDs. To address these challenges, we conducted a cross-sectional study among 6–18 year-old Arab Kuwaiti school children, to identify factors that may contribute to the development of childhood overweight and obesity.

## Subjects and Methods

### Design

A cross-sectional study was conducted in a national sample of 6,574 Kuwaiti boys and girls aged 6–18 years between September 2012 and June 2013. The sampling methodology is described in detail in our previous publication (4). Briefly, the primary sampling unit was the school and the sampling frame included primary, intermediate and secondary schools for each gender in all six governorates in the State of Kuwait. The study sample was obtained by stratifying school children based on governorate, grade and gender. A two-stage stratified random sampling technique was used to select the sample. The study protocol was reviewed and approved by the Scientific Advisory Board and Ethical Review Committee of the Dasman Diabetes Institute in Kuwait City, Kuwait, and the study was conducted in collaboration with the Ministry of Education. Study procedures were explained to the school administration and teaching staff who contacted the students' parents to obtain an informed and signed written parental consent and the student's assent.

### Anthropometric Measurements

Anthropometric measurements included body weight, height and waist-circumference (WC). Height and weight were measured with the participants being bare-footed and wearing light indoor clothing using calibrated portable electronic weighing scales (Seca, GmbH and Co., Hamburg, Germany) and portable inflexible height measuring bars, respectively. WC was measured using a constant tension tape at the highest point of the iliac crest on the mid-axillary line at the end of a normal expiration with arms relaxed at the sides. Body mass index (BMI) was calculated by dividing body weight (kg) by height squared (m). Weights were classified according to the BMI percentile charts for age and gender by the Centers for Disease Control and Prevention (CDC) (8), as underweight (BMI <5th percentile), normal weight (5th ≤ BMI <85th percentile), overweight (85th ≤ BMI < 95th percentile) and obese (BMI ≥ 95th percentile). Our research team was specifically trained in making these anthropometric measurements. Measurements were carried out in accordance with the standard operating procedures that were specifically developed for the study.

### Questionnaires

The study questionnaire was divided into two-sections. The first section, completed in the school by the study team, included general demographic information, and anthropometric measurements and the second section, completed by the parents, was intended for collecting information with regard to family demographics, socio-economic status, breastfeeding practices of the mother, birth history, eating habits, physical activity, and other health behaviors. In the eating habits section, the parents were asked how many times per week the child typically consumed fruit, vegetables, soft drinks and fast foods (burger, shawarma, etc.). In the sedentary behaviors section, the child was asked to state the average number of daily hours spent watching television and/or playing video and computer games.

### Statistical Analyses

Data completeness and accuracy were verified. Descriptive statistics were performed and presented as means and standard deviation (±/SD) for continuous variables or as numbers and percentages for nominal/categorical variables. Student's *t*-test and chi-squared test were used to evaluate differences between continuous and categorical variables, respectively. Logistic regression analysis was performed to estimate odds ratios (ORs) and to examine the predictive effect of each factor on obesity risk. ORs and their 95% confidence intervals (95% CI) for associated factors were estimated. Research Electronic Data Capture was used for data collection and data management (9). All statistical assessments were two-sided and considered to be statistically significant at *p*-values <0.05. Data analysis was performed using SAS (version 9.4; SAS Institute, Cary, NC, USA).

## Results

### Population Characteristics

Socio-demographic, lifestyle, and anthropometric characteristics of the study population are shown in Table 1. Among the 10,707 students approached, a total of 6,574 (61.4%) children and adolescents were recruited for the study; the majority were girls (3,973; 60.4%). The mean age of the study population was 12.0 ± 3.4 years (boys, 11.8 ± 3.3 years; girls, 12.1 ± 3.5 years). The study participants were stratified into three age groups: 6–10 years (35.2%), 11–15 years (45.7%) and 16–18 years (19.1%). Compared with girls, boys had a greater mean BMI (23.5 vs. 22.8 kg/m^2^; *p* < 0.0001) and WC (77.9 vs. 74.3 cm; *p* < 0.0001). Compared with girls, boys also had significantly higher intakes of fast food and soft drinks and a significantly lower intake of vegetables (Table 1). Middle Eastern culture allows more freedom and eating out behavior for boys than girls

**Table 1:** Demographic, lifestyle and anthropometric characteristics of the Kuwaiti students stratified by gender.

**Parameter**	**Kuwaiti students (6–18) years**		
	**Boys *n* = 2,601**	**Girls *n* = 3,973**	***P*-value trend**
Age (years)	11.8 ± 3.3	12.1(3.5)	0.0007
Height (cm)	150.6 ± 17.9	145.9 ± 14.2	<0.0001
Weight (kg)	55.9 ± 25.4	50.0 ± 19.6	<0.0001
Waist (cm)	77.9 ± 17.9	74.3 ± 14.5	<0.0001
BMI (kg/m^2^)	23.5 ± 7.1	22.8 ± 6.5	<0.0001
**Age group**, ***n*** **(%)**			
6–10	900 (34.6)	1,414 (35.6)	<0.0001
11–15	1,301 (50.0)	1,705 (42.9)	
16–18	400 (15.4)	854 (21.5)	
**Birth weight (kg)**			
Low (<2.5)	321 (12.3)	530 (13.3)	0.0625
Normal (2.5 - <4)	2,032 (78.1)	3125 (78.7)	
High (≥4)	248 (9.5)	318 (8.0)	
**Breastfeeding (months)**			
<2	1124 (43.2)	1,637 (41.2)	0.1953
2–6	566 (21.8)	926 (23.3)	
>6	911 (35.0)	1,410 (35.5)	
**Mother age at pregnancy (years)**			
<24	735 (28.3)	1,139 (28.7)	0.0035
24–30	875 (33.6)	1,470 (37.0)	
≥30	991 (38.1)	1,364 (34.3)	
**Mother's working status**, ***n*** **(%)**			
Working	1,354 (53.4)	2,063 (52.6)	0.5285
Not working	1,184 (46.7)	1,863 (47.5)	
**Family size**			
≤6	713 (27.4)	1,074 (27.0)	0.7348
>6	1,888 (72.6)	2,899 (73.0)	
**Mother education**, ***n*** **(%)**			
Illiterate (no schooling)	37 (1.3)	86 (1.9)	0.0734
1–12 years	1,713 (58.2)	2,575 (56.8)	
>12 years	1,196 (16.0)	1871 (41.3)	
**Family income (dinar kuwaiti)**, ***n*** **(%)**			
<800	498 (23.6)	735 (23.6)	0.9994
801–2000	1,183 (56.1)	1,750 (56.2)	
Above 2000	427 (20.3)	631 (20.3)	
Fruit (times/week)	3.4 ± 2.2	3.3 ± 2.2	0.1071
Vegetable (times/week)	3.9 ± 2.5	4.1 ± 2.7	0.0196
Fast-food (times/week)	4.2 ± 3.1	3.7 ± 3.3	<0.0001
Drinks (times/week)	5.8 ± 3.2	5.3 ± 3.2	<0.0001
TV & Video (hours/week)	9.4 ± 3.6	9.0 ± 3.6	0.0026

*BMI, body mass index; Waist, waist-circumference. Income: students average monthly household income for the last 12 months. Data are means ± SD unless noted otherwise*.

### Body Weight

**Figure 1:**
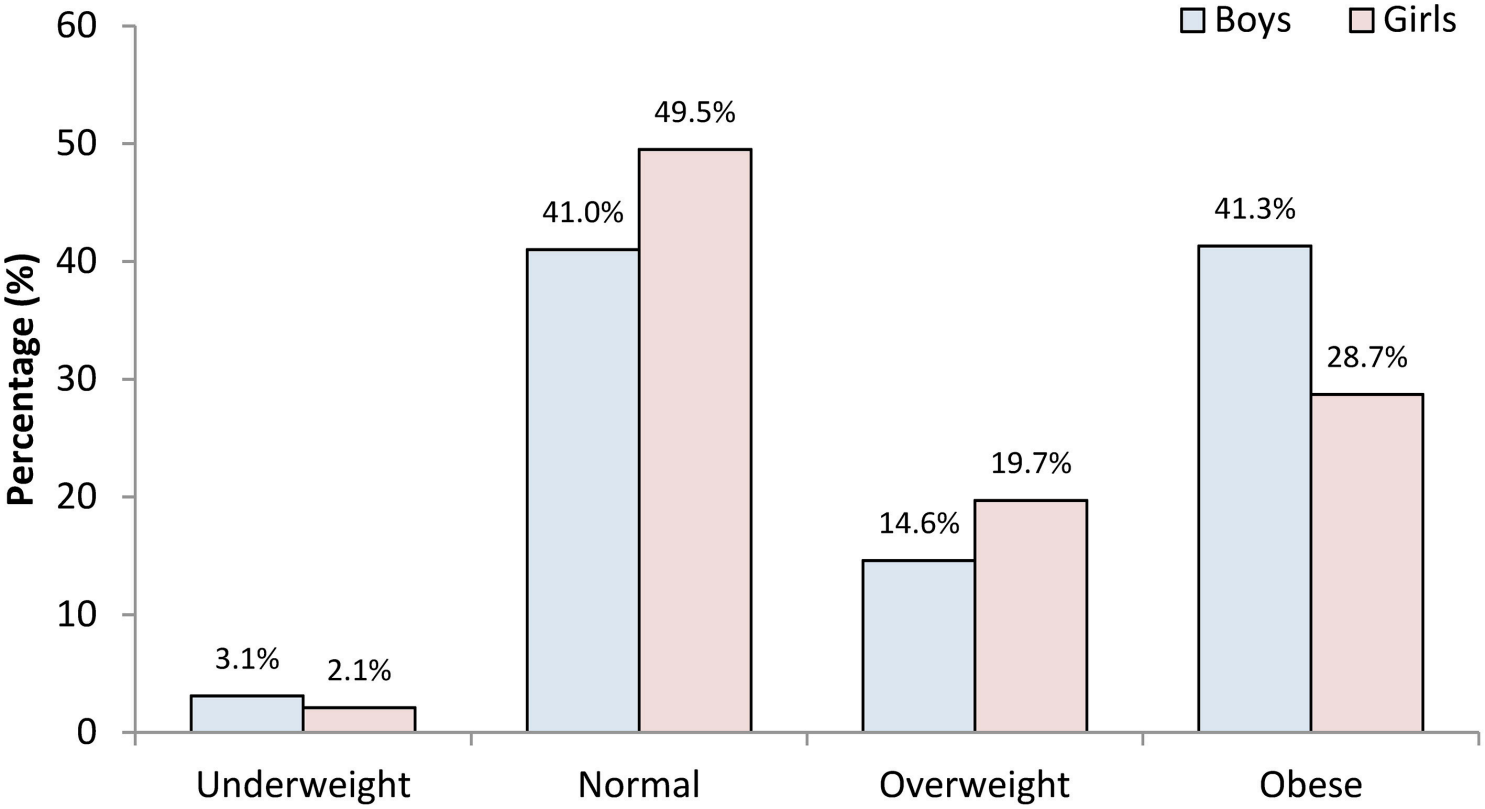
The prevalence of childhood underweight, normal weight, overweight and obesity among Kuwaiti Arab children and adolescent boys and girls aged 6–18 years.

**Figure 2:**
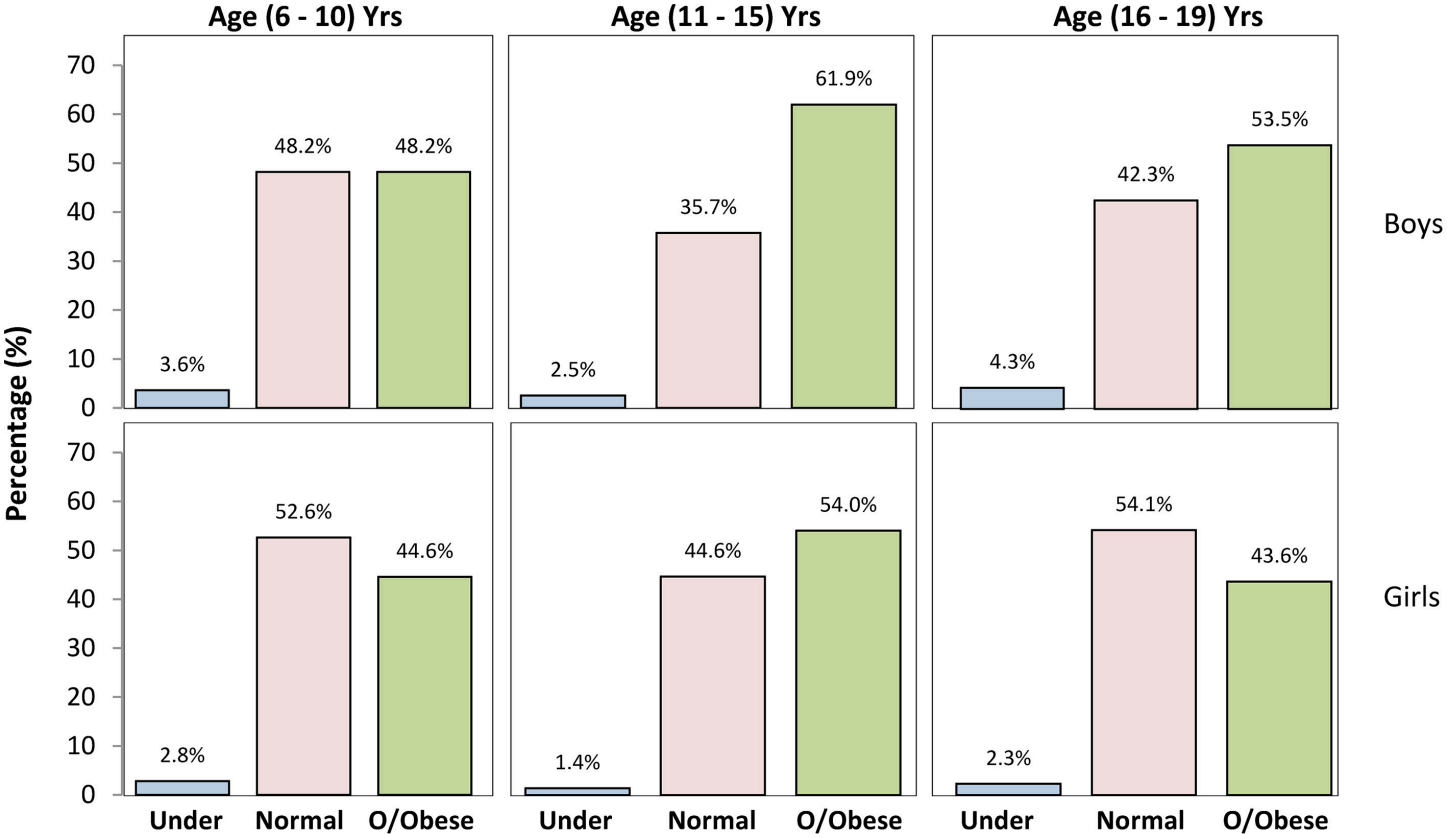
The prevalence of underweight, normal weight and combined overweight and obese (o/obese) by age group and gender among Kuwaiti students.

The prevalence of underweight, normal weight, overweight, and obesity among the entire sample was 2.5, 46.1, 17.7, and 33.7%, respectively. With regard to the overall gender differences, more girls than boys were proportionately overweight (19.7 vs. 14.6%). In contrast, 41.3% of boys were obese compared with 28.7% of the girls (Figure 1). The gender-specific distribution of BMI across different age groups (Figure 2) revealed a higher prevalence of underweight boys in all age groups compared with girls; at the same time, boys were comparatively more obese in all age groups (Table 2). In both genders, 11–15-year olds had the highest prevalence of overweight and obesity of all age groups examined.

**Table 2:** Prevalence of overweight and obesity among children and adolescents stratified by gender and age.

**Age, years**	**Weight status**	**Gender**		***P*-value trend**
		**Male (%) *N* = 2601**	**Female (%) *N* = 3973**	
6–10 (*n* = 2314)	Underweight	32 (3.6)	39 (2.8)	0.0006
	Normal weight	434 (48.2)	744 (52.6)	
	Overweight	114 (12.7)	231 (16.3)	
	Obese	320 (35.6)	400 (28.3)	
11–15 (*n* = 3006)	Underweight	32 (2.4)	24 (1.4)	<0.0001
	Normal weight	464 (35.7)	760 (44.6)	
	Overweight	207 (15.9)	374 (21.9)	
	Obese	598 (46.0)	547 (32.1)	
16–18 (*n* = 1254)	Underweight	17 (4.3)	20 (2.3)	<0.0001
	Normal weight	169 (42.3)	462 (54.1)	
	Overweight	58 (14.5)	179 (21.0)	
	Obese	156 (39.0)	193 (22.6)	

### Factors Associated With Obesity

Based on univariate logistic regression analysis, Table 3 illustrates the estimated ORs of the association between obesity and potential risk factors. Male gender was significantly associated with obesity (OR = 1.35). Age was a strong predictor of obesity; participants aged 11–15 years had a 58% increased risk of obesity as compared with those aged <11 years (OR = 1.58). Birth weight was also strongly associated with obesity; children born with weights >4 kg (8.6%) were more likely to develop obesity as they grew older (OR = 2.1). There was also a significant difference between participants whose mothers were employed vs. those who were unemployed (*P* < 0.0085). Our findings also indicated a strong correlation between childhood obesity and maternal age at pregnancy (OR = 1.24).

**Table 3:** Estimated odds ratios (OR) for obesity by associated risk factors.

**Risk factors**	**Obesity**	**Univariate logistic regression**	**Step-wise logistic regression**	***P*-value**
	**Prevalence, %**	**OR (95% CI)**		
**Age (years)**				
06–10	46.0	1	1	<0.0001
11–15	57.4	1.58 (1.42–1.76)	1.59 (1.42–1.78)	
16–18	46.7	1.03 (0.90–1.18)	1.06 (0.92–1.22)	
**Gender**				
Girls	48.4	1	1	<0.0001
Boys	55.9	1.35 (1.22–1.49)	1.29 (1.16–1.43)	
**Mother's working status**				
Not working	49.5	1	1	0.0006
Working	52.7	1.14 (1.04–1.26)	1.20 (1.08–1.33)	
**Mother age at pregnancy**				
<24	48.6	1	1	0.0016
24–30	51.0	1.10 (0.97–1.24)	1.11 (0.98–1.26)	
≥30	53.9	1.24 (1.10–1.40)	1.26 (1.11–1.43)	
**Birth weight (kg)**				
Low (<2.5)	46.9	1	1	<0.0001
Normal (2.5 – <4)	50.6	1.16 (1.00–1.34)	1.25 (1.07–1.46)	
High (≥4)	65.0	2.11 (1.69–2.62)	2.28 (1.81–2.86)	
**Breastfeeding (months)**				
<2	51.1	1	–	−
2–6	49.7	0.94 (0.83–1.07)		
>6	52.7	1.07 (0.95–1.19)		
**Mother education, %**				
>12 years	52.0	1.12 (0.97–1.28)	–	−
1–12 years	51.5	1.10 (0.96–1.25)		
Illiterate—no schooling	49.3	1		
**Family size**				
>6	50.3	1	1	0.0106
≤ 6	54.1	1.16 (1.04–1.30)	1.16 (1.04–1.30)	
**Income, kuwaiti dinar**				
<800	51.5	1	–	−
800–2000	52.2	1.03 (0.87–1.23)		
>2000	51.1	0.99 (0.84–1.16)		
**Fruits/week**				
≤ 1	51.6	1	–	−
>1	50.6	0.96 (0.85–1.09)		
**Vegetable/week**				
≤ 1	51.5	1	–	−
>1	50.8	0.97 (0.85–1.11)		
**TV and video (hours/day)**				
<1	51.0	1	–	−
1–2	50.0	0.92 (0.82–1.04)		
>2	53.7	1.11 (0.99–1.24)		
**Sporting/(hours/day)**				
≤ 1	51.5	1	–	−
>1	50.1	0.95 (0.80–1.11)		

*For forward stepwise multivariate logistic analyses, values were adjusted for all significant parameters in univariate analysis. Sporting; physical activities e.g., walking, football, running, etc*.

To analyze this relationship in more depth, we also investigated the correlation between duration of breastfeeding and maternal employment. As shown in Supplementary Figure 1A, a highly significant negative association between duration of breastfeeding and maternal employment was found. In addition, as shown in Supplementary Figure 1B, we found a significant difference in high birth weight (>4 kg) across maternity age categories at pregnancy.

In univariate analysis, there were no significant statistical associations between obesity and maternal education, duration of breastfeeding, family income, physical activity, fast food intake, or fruit and vegetable intake for both males and females (data not shown).

Multivariate logistic regression analysis (Table 3) showed that the following factors were strongly associated with obesity: male gender, older child age, smaller family size, high birth weight, maternal employment, and advanced maternal age at pregnancy. Children born to mothers who were aged >30 years at pregnancy and who were currently employed were 19 and 26% more likely to be obese, respectively. With regard to gender, boys had a 29% higher risk of obesity than girls. Similarly, children whose families had fewer than six members were at a 17% higher risk of being obese. Children with birth weight above 4.0 kg were 2.3 times more likely to be obese. Age-and gender-adjusted least squares means of maternity age and birth weight were significantly associated with both overweight and obesity in children (*P*-trend <0.0001).

## Discussion

Our findings suggest that childhood obesity is significantly related to age, male gender, birth weight, family size, maternal employment and maternal age at pregnancy. In both genders, age was a highly significant predictor of obesity, with the highest prevalence found in children aged 11–15 years. Compared with students aged 6–10 year obesity was 58% more common among those aged 11–15 years. We believe that the pubertal status of children in the 11–15 years age group may have influenced their body weight. This may also explain, at least in part, the higher prevalence of obesity in this group compared with that of the those in the 6–10 years age group (10). A large population-based longitudinal study has shown that obesity occurred early in life between 2 and 6 years in life and subsequently continued at a lower but still positive rate, which led to a greater degree of obesity to persist into adolescence, findings similar to ours (11). This observation is consistent with a previous study conducted in Kuwait (12). Nevertheless, we observed that in both genders, compared with children aged 11–15 years, overweight and obesity was lower in those aged 16–18 years. This decline in overweight and obesity with age may be explained by the rapid increase in the height of participants in this stage of life (13). Moreover, as children gain mental maturity and their self-esteem becomes more tied to their body image, they maybe more motivated to change their lifestyle and eating habits.

Gender was also found to be associated with obesity at certain ages. Major differences were observed in the 11–15 years age group; boys were at a 30% greater risk of obesity than girls. This finding is consistent with that of previous reports from other countries in the surrounding region, such as Qatar and Lebanon (14, 15). This gender difference in obesity observed in the 11–15 years age group, persisted, although the prevalence was much lower, in the 16–18 years age group. Different eating patterns and food preferences in boys compared with girls may explain this observation. Other factors may explain the discrepancy in this finding as males tend to be masculine and consume food with high dense caloric content compared to female.

Maternal employment appears to be a significant predictor of childhood obesity in Kuwait. Our study indicated that children of mothers working full-time had higher BMIs and were more likely to be obese compared with children whose mothers were unemployed. Several factors may explain how maternal employment is associated with overweight and obesity in childhood. For instance, working mothers could be pressed for time, which might cause them to substitute healthy, home-cooked meals for fast food and/or restaurant meals. Given the fact that many families in Kuwait have hired domestic help, a lack of supervision at meal times might also be associated with childhood obesity. Children of mothers who worked more hours per week over the child's life (16) and who had higher work intensity in the period after the child's birth and before the child started school (17), were shown to have an increased likelihood of becoming overweight or obese later in childhood.

At present, the mechanism by which maternal employment influences childhood obesity is unknown. However, after examining the data in more depth, we found a highly significant association between duration of breastfeeding and maternal employment, wherein maternal employment was associated with shorter duration of breastfeeding. This may indicate that supplementary feeding at an early age is a possible pathway through which children may gain weight. Therefore, we recommend that communities should provide necessary support for working mothers in the upbringing of their children so that their children's health is not compromised. Policies that support extending maternal leave may contribute to a reduction in childhood obesity rates.

Previous studies have shown that advanced maternal age at pregnancy is associated with increased rates of complications such as cesarean delivery and adverse neonatal outcomes, as well as an increased risk of gestational diabetes mellitus (GDM), preeclampsia toxemia, preterm delivery and neonatal intensive care unit admission (18, 19). Our study indicates that in addition to these outcomes, pregnancy after 30 years of age is associated with a higher prevalence of obesity among schoolchildren and adolescents. This could be due to the fact that pregnant women over 30 years old tend to have a higher maternal BMI, which is in turn associated with childhood obesity. Maternal obesity in early pregnancy has been shown to be associated with childhood obesity and to double the risk of child obesity at 2–4 years of age (20).

Birth weight is a strong predictor of child BMI (21), and this relationship was confirmed in our study, which showed that children with a high birth weight (>4.0 kg) were 2.3 times more likely to develop obesity (*p* < 0.0001). Our results also revealed a significant difference in birth weight above 4.0 Kg across maternity age at pregnancy, as pregnant women at aged >30 years tend to give birth to babies with high birth weight. Several studies have shown that higher birth weight predicted an increased risk of overweight in adolescence (11, 22) and was positively associated with adult obesity (23). Birth weight may also be a strong indicator of GDM. It is well known that children born out of pregnancies complicated by GDM are at a risk of developing obesity in childhood (24) and that maternal obesity is associated with child birth weight above the 90th percentile (25).

In our study, children whose families had fewer than six members were significantly more likely to be obese. The average family size has been decreasing worldwide, and a number of studies have indicated that in smaller families, parents tend to pay more attention to their children and their dietary habits. Family size may also be an indicator of improved socio-economic status.

This study did not identify any association between breastfeeding and childhood/adolescent obesity. This finding is in agreement with a previous study from Kuwait, which reported that neither breastfeeding nor duration of breastfeeding was associated with obesity in children aged 3–6 years (26). Surprisingly, lifestyle factors found to be associated with childhood obesity in other studies, such as television watching and physical inactivity, were not shown to be risk factors for obesity in our sample of Kuwaiti children. Similarly, our data did not establish an association between maternal education and childhood obesity. This finding is consistent with other similar studies on Arab populations (27, 28).

As any other cross-sectional study, our survey had limitations with regard to the study design. The cross-sectional nature of our study made it impossible to determine any temporal relationship between the prevalence of obesity and possible associated etiological factors. A lifestyle intervention among children in the study sample who were found to be either overweight or obese is currently underway. We also plan to prospectively follow this cohort of children to determine causality and the directional effect of the various factors contributing to childhood overweight and obesity. A second study limitation was the lack of information collected about the students' mothers regarding the prevalence of GDM, maternal obesity or gestational weight gain, all of which have been associated with childhood obesity in previous research (29, 30).

Finally, we know that dietary and activity patterns established early in life persist later in life, highlighting the importance of early intervention to develop and maintain health-promoting behaviors and healthy weight throughout life (31). Understanding risk factors and how they interact is important to inform interventions that aim to prevent obesity in early childhood (30).

## Conclusion

In conclusion, this study provides knowledge of and insight into the risk factors related to the alarmingly high prevalence of childhood obesity in Kuwait. Interventions that can successfully alter the trajectory toward overweight status among high-risk children are critical if we have to effectively address this public health crisis (30, 32). Experience in several other countries has shown that successful behavior change during childhood can be achieved through a combination of population-based measures implemented both at the national level and as part of local school and community programs (33). Special attention should be placed on women with advanced maternal age, and support should be provided to working mothers to ensure the health and well-being of their children.

## Author Contributions

NE: the principal Investigator, participated in the conception, and overall supervision of the study, handled data management, data analysis and interpretation, and wrote the manuscript; AB: a co-investigator, and participated in the study conception; MA: a co-investigator, and participated in critical revision of the manuscript; AS: a co-investigator, participated in data interpretation and critical revision of the manuscript. All authors have read and approved the final version of the manuscript.

### Conflict of Interest Statement

The authors declare that the research was conducted in the absence of any commercial or financial relationships that could be construed as a potential conflict of interest. The handling Editor declared a shared affiliation, though no other collaboration, with the authors.

## Supplementary Material

Supplementary Figure 1 **(A)** age-gender adjusted least square means of duration of breastfeeding according to the maternal employment status. **(B)** frequency of high birth weight across maternity age at pregnancy.
