# Supplementary figures and images for: The Complex Etiology of Childhood Obesity in Arabs Is Highlighted by a Combination of Biological and Socio-Economic Factors
Source: Front Public Health. 2019 Apr 2;7:72. doi: 10.3389/fpubh.2019.00072 (PMC6455072; doi:10.3389/fpubh.2019.00072)

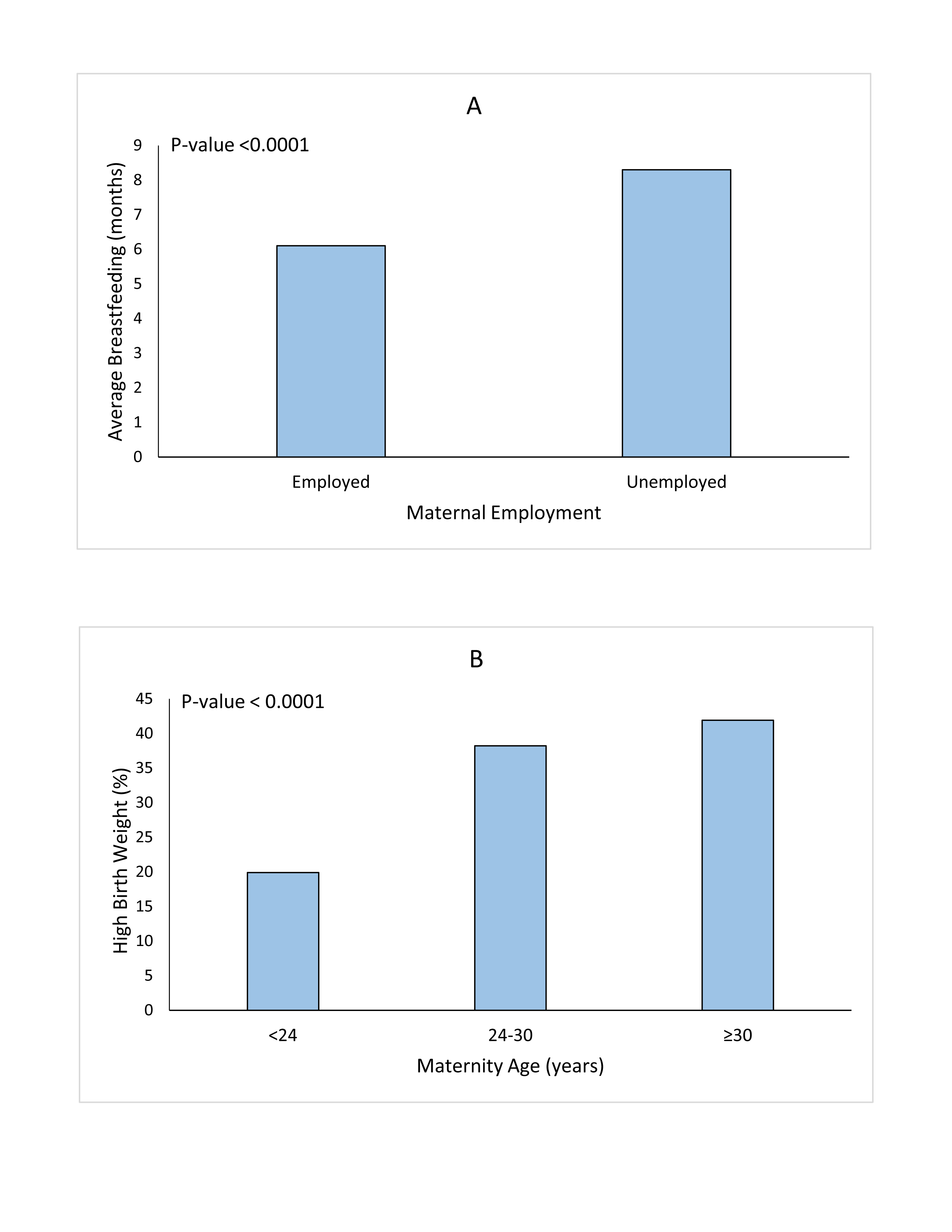

Supplement: Supplementary Figure 1 — (A) age-gender adjusted least square means of duration of breastfeeding according to the maternal employment status. (B) frequency of high birth weight across maternity age at pregnancy. [file Image_1.TIFF]
